# Sleep quality and the biological stress system during an internet-based intervention for major depressive disorder

**DOI:** 10.1016/j.cpnec.2025.100314

**Published:** 2025-08-12

**Authors:** Sebastian Laufer, Johannes Bohn, Sinha Engel, Hannah Klusmann, Nadine Skoluda, Urs M. Nater, Christine Knaevelsrud, Sarah Schumacher

**Affiliations:** a Clinical Psychological Intervention, Department of Education and Psychology, Freie Universität Berlin, Germany Schwendener Straße 27, 14195, Berlin, Germany; b Clinical Psychology and Psychotherapy, Institute for Mental Health and Behavioral Medicine, Faculty of Health, HMU Health and Medical University, Olympischer Weg 1, 14471 Potsdam, Germany; c Department of Clinical Psychology and Health Psychology, Faculty of Psychology, University of Vienna, Austria, Liebiggasse 5, 1010 Vienna, Austria; d Research Platform the Stress of Life (SOLE) – Processes and Mechanisms Underlying Everyday Life Stress, Austria; e University of Hildesheim, Institute for Psychology, Department of Experimental Psychopathology, Universitätsplatz 1, 31141 Hildesheim, Germany

**Keywords:** Alpha-amylase, Cortisol, Depression, Sleep, Internet-based intervention, Psychotherapy

## Abstract

**Introduction:**

Poor sleep quality is a persistent and debilitating symptom of major depressive disorder (MDD), with dysregulations in the biological stress system constituting a potential underlying physiological mechanism. Accordingly, a psychotherapeutic intervention may affect the interplay between sleep quality, MDD and the biological stress system.We examined how basal cortisol, and alpha-amylase levels correspond to perceived sleep quality during an internet-based intervention for MDD. Furthermore, we investigated how changes in sleep quality during the intervention relate to changes in these biological stress system markers. We hypothesized that: 1) short-term and long-term sleep quality would improve during the intervention, 2a) across assessment time points, poor sleep quality would be associated with higher cortisol and alpha-amylase concentrations, and 2b) pre-to-post intervention improvements in sleep quality (treatment response) would be associated with pre-to-post decreases in both biological markers, compared to non-response.

**Methods:**

We analyzed forty-one participants (age: 35 ± 12y; females: 82.6 %) suffering from mild to moderate MDD. The cognitive behavioral internet-based intervention consisted of seven weekly writing-based modules with individualized feedback. Participants collected 12 saliva samples at home over two consecutive weekdays at pre-, mid-, and post-intervention. Outcome parameters of the cortisol and alpha-amylase diurnal profiles were the awakening responses, the total diurnal output, and the diurnal slopes. Self-reported sleep quality was retrospectively assessed for the night before (short-term) and for the two-week period preceding saliva collection (long-term). Treatment response was determined using the reliable change index of the pre-to-post, two-week sleep quality difference scores. Hypotheses 1 and 2a were tested using random intercept hierarchical linear models, Hypothesis 2b was tested using linear regressions with age, biological sex, BMI and medication use on the day of sampling as covariates.

**Results:**

Long-term sleep quality increased significantly from pre-to post-intervention (d = 0.78; p < 0.001), partially confirming Hypothesis 1. Contrary to the expected effect of Hypothesis 2a, poor long-term sleep quality at pre-intervention was associated with a blunted cortisol awakening response (CAR; p < 0.05). Post-hoc analyses showed an association of pre-to-post CAR changes and pre-intervention sleep quality (p < 0.01) indicating that individuals with higher pre-intervention sleep problems, on average, exhibited a pre-to-post increase in the CAR. The responder analyses showed that individuals with a marked pre-to-post sleep quality increase (i.e., responders) showed a higher increase in the CAR, compared to non-responders (p < 0.05), which again ran contrary to the effect proposed in Hypothesis 2b.

**Discussion:**

Prior to psychotherapeutic treatment MDD patients with poor sleep quality showed a blunted CAR, pointing to hypocortisolemia in these individuals. Furthermore, intervention-induced changes in sleep quality may lead to a normalization of the CAR.

## Highlights

• Long-term sleep quality improved after the IBI for mild to moderate depression, while short-term sleep quality did not change.
• Poor long-term sleep quality was associated with a blunted cortisol awakening response (CAR) at pre-intervention.
• Pre-to-post intervention long-term sleep quality improvements (treatment response) were associated with a pre-to-post increase in the CAR.

## 1 Introduction

Poor sleep quality is one of the most reported and persistent residual symptoms in major depressive disorder (MDD; [1,2]), with most patients reporting insomnia symptoms (88 %), but hypersomnia also affecting roughly one third [3]. Notably, this overlap in prevalence percentages indicates that a significant number of MDD patients experience both insomnia at night and hypersomnia during the day. Concurrently, patients suffering from insomnia have an increased risk to develop MDD [4], and self-reports of sleep disturbances are significantly correlated with suicidal ideation, suicide attempts, and suicide [5], illustrating the debilitating consequences of poor subjective sleep quality on mental health and quality of life.

Several underlying biological mechanisms have been proposed as mediators between sleep quality and MDD, ranging from immune system dysregulation (e.g., increased interleukin-6 (IL-6) and C-reactive protein levels) to abnormalities in monoamine neurotransmitter regulation (for an overview, see Ref. [6]). One hypothesized pathway explaining the link between poor sleep quality and MDD is a dysregulation of the biological stress system consisting of the hypothalamus pituitary adrenal (HPA) axis, and the autonomous nervous system (ANS). Stress has been linked to both chronic insomnia and MDD, as evidenced by self-reports [7,8] and similar patterns of biological dysregulation. These include an elevated cortisol awakening response (CAR) and a flattened diurnal cortisol slope, both of which point to HPA axis hyperactivity in both disorders [[9], [10], [11]]. However, findings are inconclusive with some researchers reporting hypocortisolemia in MDD, including studies demonstrating a blunted CAR, which suggests HPA axis hypoactivity (for an overview, see Ref. [12]). Lastly, dysregulation of the stress system may also be associated with the aforementioned increased immune marker levels observed in MDD and insomnia patients, as various studies have demonstrated an interplay between the stress-induced release of cytokines and activation of the biological stress system in MDD [13]. Hence, investigating the association between sleep quality, HPA axis, and ANS activity in MDD seems worthwhile.

Regarding ANS regulation, as measured through salivary alpha-amylase (sAA), results for insomnia and MDD seem to diverge, as poor sleep quality and chronic insomnia have been associated with decreased levels of basal sAA activity [14,15], while in MDD sAA activity seems to be increased [[16], [17], [18]] and may serve as predictor of non-response to psychotherapeutic interventions [19]. One study assessing commonalities in ANS regulation between MDD and insomnia using heart rate variability (HRV) found no evidence of similar dysregulation patterns in the two disorders, compared to a healthy control group [20]. However, this study focused solely on the commonalities and differences between chronic insomnia and MDD. Consequently, the relationship between ANS regulation and sleep quality within MDD remains understudied, even though the common co-occurrence of MDD and chronic insomnia suggests such an interaction.

A study investigating the link between sleep quality and cortisol in a clinical sample reports that treatment resistant MDD patients with insomnia showed a lower CAR, compared to healthy controls with good and poor sleep [21]. Moreover, sleep quality and the CAR were both correlated with depression severity. Other studies in subclinical samples report moderating and mediating effects of sleep parameters on the association between HPA axis [22], or ANS [23] markers and depressive symptoms. Vargas et al. (2016) report that in healthy individuals who showed a correspondence between higher depressive symptoms and a shorter total sleep time, depressive symptom severity was correlated with lower waking cortisol and an increased CAR. Additionally, da Estrela et al. [23] showed in a moderated mediation analysis that mothers taking care of adolescents with developmental disorders were more likely to report poorer sleep quality compared to mothers taking care of healthy adolescents, and that this association was moderated by a lower HRV. This in turn was associated with higher depressive symptoms. The authors state that their findings may be an indication of hyperarousal of the ANS in chronically stressed individuals. Taken together, this indicates an association between sleep quality, stress system regulation, and depressive symptomatology, but that the direction of this effect (i.e., hyperactivity vs. hypoactivity) remains unclear.

Lastly, it has been shown that psychotherapeutic interventions may affect stress system dysregulations in MDD (e.g., Ref. [24,25]). Furthermore, research on sleep deprivation therapy as an add-on to conventional cognitive behavioral psychotherapy in depression showed that while there was no beneficial effect of sleep deprivation on depressive symptomatology, there was an effect on pre-stimulation cortisol and cortisol reactivity to a clomipramine challenge test [26]. More specifically, unstimulated plasma cortisol levels decreased from pre-to post-intervention in MDD patients undergoing serial sleep deprivation therapy, while cortisol reactivity increased, indicating an inhibiting effect on HPA axis activity. This suggests that changes in sleep patterns may interact with general treatment effects regarding stress system regulation over the course of psychotherapeutic interventions for MDD. Unfortunately, the authors only assessed overall depression severity but not sleep quality specifically. Hence, it remains unclear, how sleep quality and HPA axis activity change throughout a psychotherapeutic intervention and to what extent these changes covary. Assuming that psychotherapeutic interventions for depression improve sleep quality, it is conceivable that stress system regulation might also be affected. Therefore, the potential interaction of intervention-induced changes in both domains should be explored.

To date, no study has examined the association of sleep quality and both arms of the biological stress system concurrently in an MDD sample. Moreover, previous studies have assessed sleep quality either retrospectively over a longer period (e.g., the past month; [21]) or a very short period (i.e., the night before sampling; [22]), but no study has assessed both periods simultaneously. Lastly, the effect of psychotherapeutic interventions for depression on the association between sleep quality and the biological stress system has not been addressed in detail.

The current study aimed to explore how sleep quality is associated with basal stress system regulation in a sample of MDD patients with mild to moderate symptoms who participated in an internet-based intervention (IBI) for MDD. Furthermore, we examined the interaction of changes in sleep quality and basal stress system regulation. Based on the results of previous research in this area, we expected mild to moderate MDD patients who report poor sleep quality to show stress system hyperactivity, resembling subclinical [22,23] and current MDD [26] more closely than treatment resistant MDD [21,27]. Regarding our analysis of changes in sleep quality during the IBI, we expected that an improvement in sleep quality would be associated with a normalization of basal stress system activity indicated by decreased levels of HPA axis and ANS markers. Accordingly, two sets of hypotheses were tested.

Hypothesis 1 stated that sleep quality would improve from pre-to post-intervention in our sample. Hypothesis 2a stated that poor sleep quality at pre-, mid-, and post-intervention would be associated with higher basal salivary cortisol (sCort) and sAA concentrations. Related to this, hypothesis 2b stated that participants whose sleep quality improved significantly from pre-to postintervention (i.e., treatment responders) would show a decrease in basal sCort and sAA concentrations (i.e., a normalization of the biological stress system), compared to participants who do not improve.

## 2 Methods

The current paper is a secondary data analysis of a study examining the effect of an internet-based randomized controlled trial for mild to moderate depression on basal sCort and sAA levels [19], which was pre-registered at the following link: https://clinicaltrials.gov/ct2/show/NCT03752853. Please refer to this publication for a more detailed account of all methods.

### 2.1 Participants

For the original study, a total of n = 42 participants were recruited between November 2018 and April 2020 as a subsample of a larger clinical trial on the effectiveness of an IBI for mild to moderate MDD [28,29]. All participants gave written informed consent and were compensated by up to 120€ for a total of three assessment time points, paid out successively over the course of the study. Inclusion criteria were at least 18 years of age, MDD diagnosis following the Structured Clinical Interview for DSM-5 (SCID-CV; [30]), no acute suicidality and a Beck's Depression Index (BDI-II; [31]) score between 14 and 28. Exclusion criteria were a history of substance abuse (except for nicotine), psychotic disorders, bipolar disorder, intellectual disability or dementia, pregnancy or nursing within the past six months, severe somatic illness within the past five years (e.g., cancer), lifetime autoimmune disease, regular intake of psychopharmacological or glucocorticoid medication except for SSRIs, which had to be held constant during the past 12 weeks before participation. Furthermore, participants working in shift work were excluded. The study was approved by the Ethics Committee of the Department of Education and Psychology at Freie Universität Berlin, Berlin, Germany (reference: 148/2017).

### 2.2 Procedure

Participants received comprehensive study information via telephone and in writing, following completion of the SCID-CV interview [30] and gave informed consent. Study materials were sent by mail to the participants’ home address. Furthermore, participants received written and video instructions on saliva collection procedures via e-mail. Saliva was collected at three assessment time points throughout the IBI (i.e., pre-, mid-, and post-intervention). Pre-intervention data collection (T0) was scheduled directly before the start of the IBI, mid-intervention data collection (T1) after completion of the fourth module of the IBI after approximately four weeks, and post-intervention (T2) directly upon completion of the IBI, after approximately seven weeks.

### 2.3 Internet-based intervention

The IBI was designed to reduce depressive symptoms. This includes sleeping difficulties, albeit these were not directly focused on. Details regarding the intervention have been published elsewhere [28,29]. In brief, we conducted a therapist-guided, asynchronous, writing-based psychotherapeutic intervention for mild to moderate depression consisting of seven consecutive modules (i.e. sessions). Each patient was assigned one therapist. Participants completed one module each week upon which they received written feedback. Psychologists were licensed psychotherapists or advanced psychotherapists in training with extensive experience in treating MDD. In case of adverse events or an increase in suicidality throughout the intervention, psychologists followed a standard operating procedure (for details see Ref. [29]).

The intervention was based on cognitive behavioral therapy for MDD. The seven treatment modules included written or audiovisual (video) information on MDD, followed by information about the task to be completed in that week. The first two modules were expressive writing tasks focusing on pathogenesis, current symptoms, and goals for the intervention. More specifically, participants were instructed to write two letters describing when and under which circumstances they first felt depressed (letter one) as well as their current symptoms and goals for the intervention (letter two). Next, participants completed two modules on behavioral activation and two modules on cognitive restructuring. During behavioral activation, participants used a weekly planning tool to schedule pleasant activities, record whether they had completed the activity and comment on how they felt while pursuing it. During cognitive restructuring, participants completed a so-called interpretation training during which they were presented with exemplary situations for which they had to choose a response typical for their way of thinking and received feedback on the cognitive biases these responses illustrated [32]. In a second step, participants used a logbook to record individual situations, their own initial thoughts and alternative interpretations. The order of these modules was pseudorandomized (for details, see Ref. [28]). The last module consisted of a third expressive writing task during which participants reflected on the intervention, their new insights and how they might prevent future depressive episodes. Participants received individualized written feedback on each of the modules and were able to contact their study therapists via direct message during the intervention in case of unclarities or technical difficulties.

### 2.4 Psychological measures

Sleep quality was assessed by self-report. Participants answered questionnaires regarding their short-term and long-term sleep quality at T0, T1, and T2. Short-term sleep quality here refers to the night before each saliva collection day, while long-term sleep quality refers to the two weeks prior to the three assessment time points (T0, T1, and T2). We are aware that this definition of long-term sleep quality rather reflects a conventional assessment period in other studies, since sleep quality shows high inter-night variability [33,34]. Therefore, all references made to the temporal dynamics of sleep quality (i.e., long-term vs. short-term) have to be understood as relating to each other, not an absolute estimate.

Short-term sleep quality assessment included the self-reported sleep duration (minutes), the total time in bed (minutes), the usual sleep time (minutes), the number of nocturnal awakenings (1, 2, 3, or more than 3 times), and the overall subjective sleep quality presented as a visual analog scale ranging from 1 (very poor) to 10 (very good). These variables were summarized to a short-term sleep quality indicator (STSQ; see section 2.6.1).

Long-term sleep quality was assessed by the German version of the Pittsburgh Sleep Quality Index (PSQI; [35]). We adapted the time frame of interest for the current study from the original past four weeks to the past two weeks. This was done as a precaution to avoid potential overlap of time periods between assessment T1 and T2.

### 2.5 Biological measures

Saliva samples were analyzed for sCort and sAA. An overview of all assessment protocol specifications can be retrieved from the Cortisol Assessment List (CoAL; [36]) in the Appendix or under https://osf.io/prt8s/. At each assessment time point (T0-T2), participants collected a total of 12 saliva samples over two consecutive weekdays. Sample collection times were directly upon awakening, 30 min after awakening, at 11:00, 14:00, 18:00 and 21:00 on two consecutive weekdays at each assessment time point. Participants recorded the exact sampling times on a protocol sheet. Saliva was collected via passive drool into Salicaps® (IBL, Hamburg, Germany) which were stored in an HDPE container. To minimize the influence of state covariates [37] on sCort and sAA, participants refrained from consuming alcohol or physical exercise 24 h before each assessment and refrained from eating, drinking (except for water), consuming caffeine, smoking, brushing their teeth, and sleeping (except upon awakening), 60 min before each sampling time point [38]. They documented any deviations from this protocol. Lastly, participants documented any medication intake on the day of sampling as well as any extraordinary events that may have caused stress (e.g., arguments, pain, small accidents, etc.). During sample collection participants kept the samples frozen at home at −18 °C. After completing the sample collection at each assessment time point, participants sent back the saliva samples by mail to the research team at Freie Universität Berlin. Upon arrival, samples were stored at −20 °C so in total, samples underwent one thawing and freezing cycle. Once data collection for all participants was completed, samples were sent to the biochemical laboratory for analysis. Saliva samples were kept frozen during transportation. All biochemical analyses were conducted at the Biochemical Laboratory of the Department of Clinical and Health Psychology at the University of Vienna, Austria. SCort was measured using a commercially available enzyme-linked immunoassay (IBL; Hamburg, Germany). SAA was assessed using an enzyme kinetic colorimetric test [39] and reagents from DiaSys Diagnostic Systems (Holzheim, Germany). Inter- and intraassay variations were below 10 %, respectively.

### 2.6 Data preparation

#### 2.6.1 Long-term and short-term sleep quality

To quantify short-term sleep quality, we derived the Short-Term Sleep Quality indicator (STSQ), based on self-reported sleep duration, total time in bed, usual sleep duration, nocturnal awakenings and subjective sleep quality of the night before each saliva collection day. Variables were averaged across the two collection days of each assessment time point (T0, T1, and T2). We then calculated sleep efficiency and sleep deficit. Sleep efficiency was defined as the ratio of sleep duration to total time in bed [40]. Sleep deficit was defined as the difference between the reported and the usual sleep duration.

Subsequently, we conducted a principal component analysis (PCA) to identify the variables most representative of the STSQ. The model was considered valid if only one component (C1) had an Eigenvalue >1 and accounted for the majority of variance (see Appendix for details). Subjective sleep quality was retained in all models as a non-replaceable indicator, given its direct relevance.

The initial full model PCA included sleep efficiency, sleep deficit, nocturnal awakenings and subjective sleep quality, which yielded a two-factor solution and was therefore rejected (41.6 % explained variance; Eigenvalues: C1 = 1.664; C2 = 1.274; C3 = 0.542; C4 = 0.520). We subsequently tested reduced models by excluding each variable in turn, except subjective sleep quality. Out of these, the best fitting model meeting all validity criteria included sleep quality, sleep deficit and sleep efficiency (55 % explained variance; Eigenvalues: C1 = 1.663; C2 = 0.808; C3 = 0.529). The final STSQ score was computed as:

STSQ=−0.4765898×sl.quality−0.6121972×sl.deficit−0.6309333×sl.efficiency

To calculate the STSQ indicator, all input variables were z-transformed. As such, the STSQ has a mean of zero which represents the sample's average short-term sleep quality. Accordingly, STSQ scores >0 represent lower than average sleep quality, while STSQ scores <0 represent higher than average sleep quality.

Long-term sleep quality was expressed through PSQI sum scores, again with higher scores indicating more severe sleep problems. Furthermore, PSQI scores were categorized into “good” and “poor” long-term sleep quality. Here, the original paper suggests a cut-off score of 5 indicating poor sleep [41]. However, the validation study of the German version suggests using a cut-off score of 6 as this significantly increases sensitivity while only marginally reducing specificity [35]. For this reason, a cut-off of 6 was employed.

Lastly, we quantified treatment response in long-term sleep quality by calculating the reliable change index (RCI) of the PSQI delta scores (PSQI_post_ – PSQI_pre_). The RCI approach was chosen, because there are no empirical cut-off values for the PSQI that would indicate clinically significant change. The reliability estimate for the PSQI was set at 0.87 [35]. Based on the RCI, participants were divided into *responders* (RCI < −1.96), and *non-responders* (RCI ≥ −1.96). Since the STSQ does not have a reliability estimate, computation of an RCI was not possible and we refrained from testing short-term sleep quality in this regard. Skewness and kurtosis were inspected for both sleep quality indicators. Here, distribution was sufficiently normal for PSQI scores, while STSQ scores showed high skewness and kurtosis values. To be able to continue interpreting the STSQ as a z-score, we avoided a transformation.

#### 2.6.2 Biological data

The data was inspected for outliers in combination with deviations from the adherence protocols (e.g., nil by mouth in the hour before sampling). In total, three analysis parameters characterizing the diurnal sCort and sAA profiles were used: the CAR or alpha-amylase awakening response (AAR), the total diurnal output and the diurnal slope. The CAR and AAR were calculated as the delta scores of the waking and the waking +30 min samples corrected for the exact time between samples. Total diurnal output was expressed as the area under the curve with respect to ground (AUC_g_; [42]) and the diurnal slope as the linear regression coefficient of the 11:00, 14:00, 18:00, and 21:00 samples. All parameters were calculated using the untransformed data first. Transformation was only considered in case of high skewness or kurtosis. This was the case for the sAA AUC_g_. For this parameter, the natural logarithm (ln) of the raw data was used.

### 2.7 Statistical analysis

#### 2.7.1 Preliminary analyses

Before conducting the hypothesis-driven analyses, three preliminary tests were conducted. First, we examined whether improvements in sleep quality were influenced by baseline sleep quality, as the IBI might have been particularly effective in participants with higher pre-intervention sleep disturbances. To this end, the variance of STSQ and PSQI scores was inspected per assessment time point. Here, one would expect variance to decrease over time if the intervention was effective only in participants with severe pre-intervention sleep disturbances. Second, we checked whether the STSQ and the PSQI questionnaires measured different constructs by computing Pearson correlations between STSQ and PSQI across time. Lastly, the stability of each sleep quality measure was examined by calculating the bivariate correlation between the three assessments, respectively.

#### 2.7.2 Sleep quality improvement

To test whether sleep quality would improve from pre-to post-intervention (hypothesis 1), we employed a random intercept hierarchical linear model approach. Two regression models were fitted with either short-term (STSQ) or long-term (PSQI) sleep quality as the dependent variable, time as the within-subject factor, and biological sex, age, and body mass index (BMI) as covariates. To control for multiple testing in this and all subsequent analyses, we conducted a Bonferroni-Holm correction. Accordingly, we report the adjusted p-values (p_adj_) in the results section. Post-hoc pairwise comparisons of the three assessment time points were performed using estimated marginal means, and effect sizes were calculated using Cohen's d [43].

#### 2.7.3 Association of sleep quality and HPA axis or ANS activity

We hypothesized that poor sleep quality would be associated with higher sCort and sAA concentrations (hypothesis 2a). Accordingly, higher scores on the STSQ and PSQI scales, indicative of poor sleep quality, should be associated with higher CAR/AAR values and a greater AUC_g_ for both biological markers. The beta coefficients representing the diurnal slopes of sCort (negative beta coefficients) were expected to approach zero, while those for sAA (positive beta coefficients) were expected to increase with higher STSQ and PSQI scores. This hypothesis was also examined using random intercept hierarchical linear models. In total, 12 regression models were calculated (three analysis parameters × two biological markers × two sleep quality estimates). The respective analysis parameter (i.e., the awakening response, the AUC_g_, or the diurnal slope) for sCort or sAA was entered as the dependent variable. STSQ or PSQI scores served as the between-subject factor, and assessment time point (T0, T1, T2) as the within-subject factor. Covariates were age, biological sex, BMI, and medication on the day of saliva sampling (yes/no). Lastly, an interaction term of PSQI or STSQ by assessment time point was added to the model. In case of a significant interaction, simple slopes per assessment time point were calculated. In case of a non-significant interaction the model was rerun, excluding the interaction term.

#### 2.7.4 Association of treatment response and change in HPA axis or ANS activity

To test whether treatment responders would show greater decreases in sCort and sAA concentrations compared to non-responders (hypothesis 2b) we calculated a total of six linear regression models (three analysis parameters × two biological markers). The RCI-based PSQI response variable was entered as the independent variable with non-responders as the reference group. The delta scores of the respective sCort/sAA analysis parameter (e.g., AUC_g-post_ – AUC_g-pre_) was entered as the dependent variable. We expected higher negative pre-to-post delta scores for the three sCort and sAA analysis parameters in responders compared to non-responders. Covariates again were biological sex, age, BMI, and medication use on the day of saliva assessment.

## 3 Results

### 3.1 Sample characteristics and preliminary analyses

Table 1 shows baseline demographic information. As can be seen, the sample was middle-aged on average and predominantly female. PHQ-9 scores indicate moderate depression on average.

**Table 1:** Baseline demographic information.

Variable		Value
N total		46
Age (M ± SD)		35.2 ± 12.1
Female n (%)		38 (82.6 %)
BMI (M ± SD)		23.6 ± 3.8
SSRI	No n (%)	44 (95.7 %)
	Yes n (%)	2 (4.3 %)
OC	No n (%)	35 (76.1 %)
	Yes n (%)	11 (23.9 %)
PHQ-9 (M ± SD)		12.8 ± 3.2

*Note.***BMI**: Body mass index; **SSRI:** Selective serotonin reuptake inhibitors; **OC:** Oral contraceptives; **PHQ-9:** Patient Health Questionnaire.

Table 2 summarizes sleep quality and biological data over all three assessment time points. As can be seen, the variance of sleep quality did not change over time, indicating that profiting from the intervention did not seem to be affected by baseline sleep quality. The correlation of STSQ and PSQI scores across all assessment time points was moderate (Pearson's r = 0.41; p < 0.001). Hence, both indices seemed to differ sufficiently from each other regarding the time frame they assessed. Lastly, correlations were moderate between assessment time points for both measures (STSQ: pre & mid: r = 0.45; p < 0.001; pre & post: r = 0.48; p < 0.001; mid & post: r = 0.58; p < 0.001; PSQI: pre & mid: r = 0.42; p < 0.001; pre & post: r = 0.46; p < 0.001; mid & post: r = 0.67; p < 0.001), indicating sufficient stability across time [44].Notably, the first row shows the number of participants over time with 89.1 % of participants completing all three assessments. Long-term sleep quality, as illustrated through the PSQI scores and the PSQI categories shows that average pre-intervention sleep quality was rather poor. During the intervention, mean PSQI scores decreased, indicating an improvement in long-term sleep quality. Since no normative data for the STSQ exist, no absolute information regarding short-term sleep quality can be provided. However, the median STSQ scores also decreased during the intervention, indicating an improvement in short-term sleep quality. Regarding the biological data, sCort levels on average stayed fairly constant across all three assessment time points while sAA concentrations on average increased from pre-to post-intervention. An overview of the analysis parameters over time can be found in the Appendix.

**Table 2:** Sleep and biological data over time.

Variable		T0	T1	T2
No. participants *n (%)*		46 (100.00)	44 (95.65)	41 (89.13)
PSQI score *M (SD)*		7.54 (2.45)	6.30 (2.69)	5.56 (2.76)
PSQI categories *n (%)*	*Good*	15 (32.61)	27 (61.36)	29 (70.73)
	*Poor*	31 (67.39)	17 (38.63)	12 (29.27)
PSQI response *n (%)*	*Resp.* *Non-resp.*			16 (39.02) 25 (60.98)
STSQ score *M (SD)*		−0.04 (1.13)	−0.01 (1.23)	0.06 (1.54)
sCort nmol/l *M (SD)*		11.19 (5.17)	10.19 (4.00)	10.70 (4.56)
sAA U/ml *M (SD)*		42.92 (35.81)	48.38 (37.31)	58.14 (60.80)

*Note.* PSQI: Pittsburgh sleep quality index; STSQ: Short-term sleep quality; sCort: salivary cortisol; sAA: salivary alpha-amylase; T0: Pre-intervention assessment time point; T1: Mid-intervention assessment time point; T2: Post-intervention assessment time point.

### 3.2 Treatment response on sleep quality

**Fig. 1:**
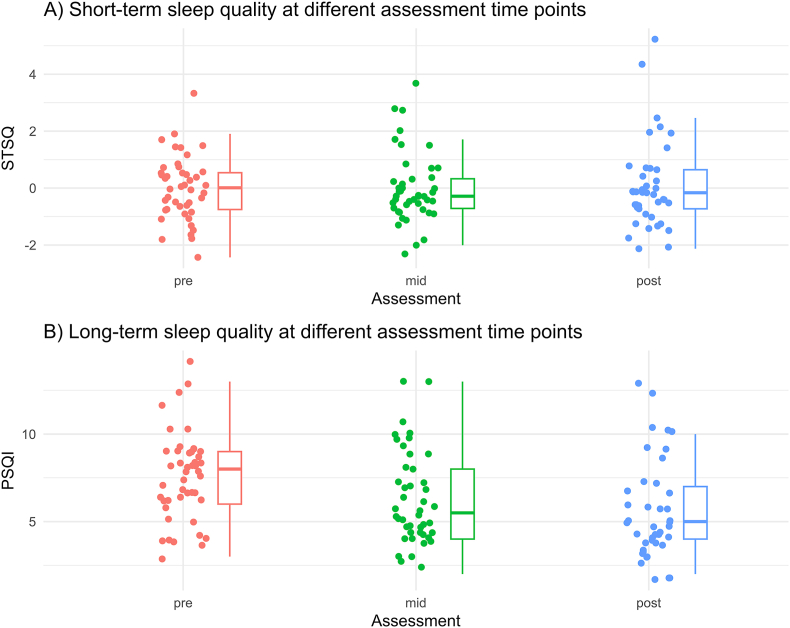
Time course of sleep quality during the internet-based intervention. *Note.***STSQ:** Short-term Sleep Quality **PSQI:** Pittsburgh Sleep Quality Index. For both indices, lower values indicate a better sleep quality.

Both sleep quality trajectories are illustrated in Fig. 1. Short-term sleep quality results did not show a significant effect from pre-intervention (T0) to mid-intervention (T1; estimate = 0.01; se = 0.20; df = 84.45; p_adj_ = 0.97) or pre-to-post-intervention (T2; estimate = 0.05; se = 0.21; df = 85.84; p_adj_ = 1.00). Results of the long-term sleep quality analysis during the intervention showed a significant effect from both T0 to T1 (estimate = −1.30; se = 0.40; df = 85.64; p_adj_ < 0.01), and from T0 to T2 (estimate = −2.04; se = 0.40; df = 85.73; p_adj_ < 0.001). Post-hoc tests showed medium effect sizes for both pairwise comparisons from T0 to T1 (d = 0.50) and T0 to T2 (d = 0.78), and a small effect size from T1 to T2 (d = 0.28). Furthermore, almost 40 % of participants experienced an improvement in long-term sleep quality based on the RCI of pre-to-post PSQI delta scores.

### 3.3 Association between sleep quality and HPA axis or ANS activity

**Fig. 2:**
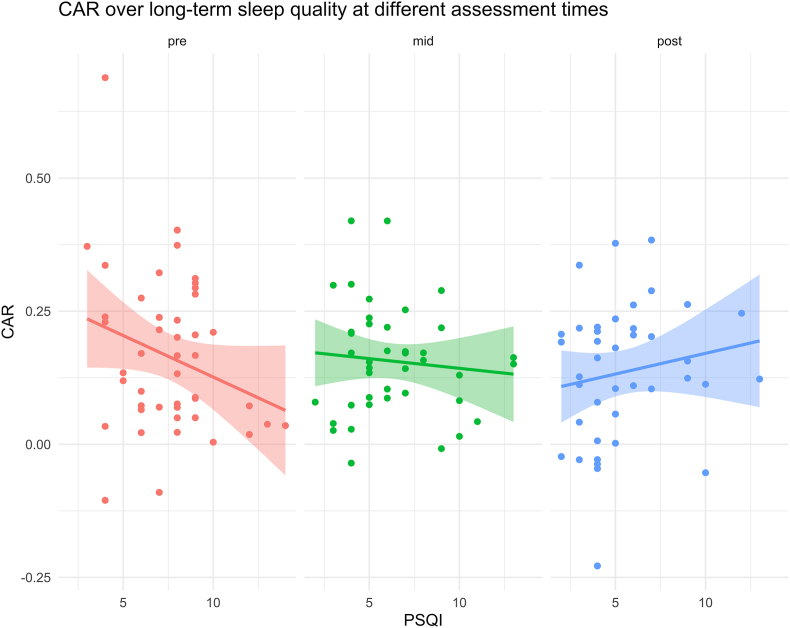
Time course of the association of sleep quality and the cortisol awakening response. Plots show the regression lines at pre-intervention (left), mid-intervention (mid) and post-intervention (right) for long-term sleep quality. *Note.***CAR:** Cortisol awakening response; **PSQI:** Pittsburgh Sleep Quality Index with higher scores indicating lower sleep quality; **pre:** Pre-assessment; **mid:** Mid-assessment; **post:** Post-assessment.

Only the CAR showed a significant association with long-term sleep quality. Taking T0 as reference, there was a significant time-by-PSQI score interaction (T2-by-PSQI: estimate = 0.039; se = 0.01; df = 92.39; p_adj_ < 0.05), indicating different associations of the CAR and long-term sleep quality across time. This effect is controlled for medication use on the day of the assessment (i.e., medication use reduced the CAR with an estimate of −0.07; p < 0.05). Simple slope analysis showed a significant association of PSQI score and the CAR at T0 (estimate = −0.02; se = 0.01; p < 0.001), while no such association was evident at T1 (estimate = −0.01; se = 0.01; p = 0.30) or T2 (estimate = 0.01; se = 0.01; p = 0.43). The effects are illustrated in Fig. 2. The effect at T0 indicates that lower sleep quality in the two weeks prior to assessment were associated with a lower CAR. No other sCort or sAA analysis parameters showed an association with long-term or short-term sleep quality.

**Fig. 3:**
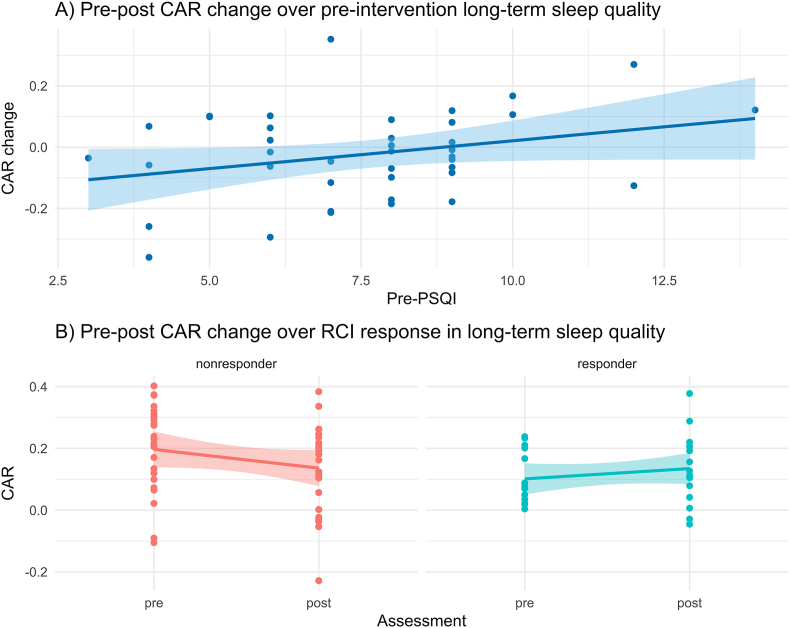
CAR change score analyses *Note.***A)** Association between changes in the CAR and pre-intervention long-term sleep quality. Higher PSQI scores indicate lower sleep quality. **B)** Association of changes in the CAR by response status based on the reliable change index with non-responders on the left (red) and responders on the right (cyan). **CAR:** Cortisol awakening response; **PSQI:** Pittsburgh Sleep Quality Index.

In a post-hoc analysis, we examined whether the non-significant associations of PSQI scores and the CAR at T1 and T2 might be due to improvements in sleep quality, particularly in participants with lower baseline sleep quality. Therefore, we regressed the pre-to-post change of the CAR on pre-intervention PSQI scores. On average, the CAR across the sample decreased (T0: mean (sd) = 0.164 (0.148); T1 mean (sd) = 0.156 (0.104); T2 mean (sd) = 0.135 (0.130)). Results showed that pre-intervention PSQI scores were significantly associated with pre-to-post changes in the CAR (estimate = 0.02; se = 0.009; df = 34; p < 0.01). Hence, participants with lower pre-intervention sleep quality showed either a higher CAR increase from pre-to post-intervention, or less of a decrease (see Fig. 3A). This effect is controlled for biological sex (estimate = 0.13; se = 0.06; df = 34; p < 0.05) and age (estimate = −0.004; se = 0.002; df = 34; p < 0.05).

### 3.4 Association of treatment response and change in HPA axis or ANS activity

Results of the responder analysis showed a significant association between response status, and pre-to-post changes in the CAR (estimate = 0.12; se = 0.04; df = 34; p_adj_ < 0.05). This indicates that participants whose long-term sleep quality improved particularly well during the intervention, showed a pre-to-post increase in the CAR, compared to participants who did not benefit as much. Again, this effect is controlled for biological sex (estimate = 0.13; se = 0.06; df = 35; p < 0.05) and age (estimate = −0.004; se = 0.002; df = 35; p < 0.05). An illustration of the main effect can be found in Fig. 3B.

## 4 Discussion

This secondary data analysis examined the relationship between sleep quality and basal stress system regulation throughout an IBI for mild to moderate MDD. In line with hypothesis 1, our findings indicate that the IBI improved long-term sleep quality. However, contrary to expectations, short-term sleep remained unchanged. Consistent with hypothesis 2a, long-term sleep quality was associated with the CAR at pre-intervention, albeit in the opposite direction of predictions with poorer sleep quality correlating with a blunted CAR and greater CAR increases from pre-to post-intervention. Likewise, pre-to-post improvements in sleep quality (i.e., treatment response) were significantly associated with increases in the CAR, which again ran contrary to the expected effect put forward in hypothesis 2b.

Regarding the IBI's effect on sleep quality, the discrepancy between improvements in long-term but not short-term sleep quality is not intuitive at first glance. However, sleep quality is a highly variable construct, and multi-night aggregate measures are usually recommended to assess it [34]. Hence, an overall effect may not become apparent in the acute assessment of sleep quality over two nights. However, the absence of an intervention effect does not seem to be due to insufficient STSQ stability, as correlations between assessment time points were comparable across sleep quality measures. Instead, participants might have distinguished between their daily fluctuations in sleep quality and overall trends.

Alternatively, mood-related self-report and memory biases may explain the divergence. Indeed MDD patients tend to misreport some sleep quality parameters like sleep onset latency or total sleep time [45] and poor sleep quality is associated with an increase in negative affective memory recall [46]. In the absence of objective sleep data, a reporting bias cannot be ruled out, warranting the need for replication by future research.

Regarding hypothesis 2 a), our results could not confirm earlier work indicating an association between an elevated CAR and depressive symptoms in individuals with shorter sleep duration [22] or ANS regulation and sleep quality [23]. They also seem to contradict a prospective study suggesting that adolescents with an increased CAR and poor sleep showed increased depressive symptoms at a two-year follow-up [47]. These discrepancies may be explained by differing sample characteristics (i.e., healthy participants vs. MDD patients) or sleep parameters (i.e., total sleep time the previous night vs. sleep quality including total sleep time among other variables).

Our results align with the findings reported by Santiago et al. [21], showing that treatment-resistant MDD patients with poor sleep exhibited a blunted CAR. The same group replicated this effect, demonstrating a blunted CAR in treatment resistant MDD, compared to mild MDD, and healthy controls [27]. Notably, the authors here showed that the mild MDD group exhibited a higher CAR, compared to healthy controls pointing to hypercortisolemia in this cohort. Since we examined mild to moderate MDD, our results do not necessarily point to a link between poor sleep quality and hypocortisolemia *per se*. It may still be the case, that the MDD patients in our sample exhibited HPA axis hyperactivity (for an overview, see Ref. [48]). However, our results suggest that poor sleep quality may interact with overall HPA axis regulation pointing to a relative blunting effect of poor sleep on the CAR, even within mild to moderate MDD. Alternatively, the effect presented by Torres and colleagues [27] might be driven primarily by sleep quality and not MDD cohort allocation, as treatment-resistant MDD patients scored significantly higher on the PSQI, compared to mild MDD patients.

Hypocortisolemia has been proposed as an endocrinological indicator of a symptom triad including high stress sensitivity, fatigue, and pain observed in a variety of psychological disorders like fibromyalgia, PTSD, and chronic pain [49]. In this context, it may arise as an adaptive response following prolonged HPA axis hyperactivity mitigating allostatic overload [50]. Our results fit in with this notion since poor sleep quality is associated with increased levels of fatigue and our sample showed markedly higher chronic stress levels, compared to a general population benchmark, as reported in a previous publication [19]. This may have contributed to MDD pathogenesis, as individuals who exhibited poor sleep quality were less able to cope with this prolonged stress exposure.

The selective intervention effect on long-term sleep quality may explain the observed association between long-term sleep quality and the CAR at pre-intervention. Since mean pre-intervention long-term sleep quality was significantly worse, compared to mid-, and post-intervention, a stronger association was evident, particularly among participants with severe sleep disturbances. Assuming that a blunted CAR only manifests in MDD patients with severely disturbed sleep, the association may diminish as sleep improves during the intervention. Accordingly, an improvement in long-term sleep quality during the intervention could lead to a normalization of the CAR, particularly in these individuals. Our post-hoc analysis supports this claim. Here, high pre-intervention PSQI scores were associated with a pre-to-post increase or less of a decrease in the CAR.

The significant association between treatment response and an increase in the CAR further supports the normalization claim stated in hypothesis 2b. To our knowledge, this is the first study linking improved sleep quality to CAR increases. If this effect is replicable, the clinical implications for MDD and other patient groups are significant. The potential to help normalize HPA axis dysregulation by psychotherapeutically increasing sleep quality may prompt practitioners to focus more on this symptom, which is highly prevalent in MDD but by no means exclusive to it. The possible long-term health benefits of this approach become evident when considering that HPA axis dysregulation has been shown to be associated with conditions like cardiovascular disease, autoimmune disorders, and diabetes [51] all of which are more prevalent in MDD [[52], [53], [54]]. Reducing HPA axis dysregulation through psychotherapeutic improvements in sleep quality could lower the risk of these adverse health outcomes not only in MDD but also other patient populations affected by poor sleep.

Contrary to our expectations, none of the sAA analysis parameters reached significance. This may have been due to the limited statistical power, or noise introduced through the moderate intra-individual stability of diurnal sAA regulation across time [39]. Moreover, the opposing ANS regulation effects of chronic insomnia (i.e., increased levels of sAA) and MDD (i.e., decreased levels of sAA) presented in the introduction might have interacted in our sample masking any clear effect. However, these assertions remain speculative at this point and require replication.

To summarize, our findings suggest the concurrent improvement of sleep quality and normalization of the CAR particularly in MDD patients with severe sleep problems following an IBI. These results indicate that psychotherapeutic interventions affect both sleep quality and its biological correlates in MDD.

## 5 Limitations and future directions

Several limitations and directions for future research should be mentioned. Firstly, this is a secondary data analysis not tailored to the research question at hand. Therefore, our results offer only preliminary evidence for an association of the CAR and sleep quality as well as an intervention effect of the IBI on long-term sleep quality and the CAR. Related to this, no claims regarding an absolute hyper- or hypoactivity of the stress system in MDD can be made. All references to these two constructs always pertain to a relative estimate within the MDD population, comparing good and poor sleepers, but not healthy control participants. However, the current results show that sleep quality might significantly account for interindividual CAR variability within MDD samples. This may help in explaining contradictory results regarding the direction of HPA axis dysregulation within this population. Future work may elucidate the potential of the CAR as an indicator of sleep problems in MDD with a larger sample size, specifically stratified according to sleep quality and MDD.

Secondly, the IBI did not address sleep problems in particular and we were not able to recruit a waitlist control group. Hence, the improvements observed in our sample regarding both, overall mood and sleep quality are not attributable to the intervention *per se* (compare Hypothesis 1) and may have occurred as a function of time. Moreover, no causal attribution can be made as to whether better sleep leads to a normalization of the CAR and the reported association between changes in the CAR and changes in long-term sleep quality (compare Hypothesis 2b) could still be a function of overall symptom improvement. However, our earlier publication on the matter did not indicate this showing no association between overall depression symptom improvement and this analysis parameter [19]. Therefore, investigating the specific effects of therapeutic attempts to improve sleep quality seems highly relevant for future intervention studies. Future work may target sleep quality more directly in a randomized controlled trial. This way, a causal link between improvements in sleep quality and a potential normalization of the CAR may be established, particularly in MDD patients with poor sleep.

Thirdly, our assessment protocol included two potential limitations as our analysis did not include objective measures of sleep quality and saliva samples were thawed during postal delivery from the participants to our facilities. This may limit the reliability of our results. First, the sole reliance on self-reports cannot control for potential reporting biases, that may have been affected by confounding factors such as overall mood. However, we chose to focus on subjective sleep quality, as this seems to affect participants’ well-being more profoundly compared to objective sleep parameters. Second, the thawing and re-freezing cycle as well as the postal delivery of the samples may have lowered sAA levels, as indicated by a recent study [55]. We cannot rule out that this may have affected our sAA results. However, other studies find sAA to be reasonably robust against temperature variations [56]. Furthermore, ensuring continuous cooling would not have been feasible in the current study without increasing participant burden significantly. Heyers et al. [55] state that the impact on analysis results is still to be determined. Therefore, one must weigh the benefits obtaining field data against the risk of significant noise introduction through logistical obstacles. Nevertheless, future work may implement a more comprehensive assessment of subjective and objective sleep parameters and circumvent thawing prior to final biochemical analysis to establish the association between sleep and biological markers of the stress system.

Finally, our sample size may have been underpowered to detect associations with other sCort or sAA analysis parameters. Future research could aim to establish potential links between sleep quality and other cortisol analysis parameters like the AUC and diurnal slope, or other biological markers of the stress system, especially those related to ANS activity. This seems highly relevant, as the HPA axis only constitutes one arm of the biological stress system and information on the interplay between both arms in MDD could prove vital to future therapeutic attempts.

## 6 Conclusion

To conclude, this study shows that subjective long-term sleep quality is associated with basal HPA axis dysregulation in MDD. Furthermore, our results show that an IBI might not only improve sleep quality over a relatively short period of time but also help to normalize dysregulations of the HPA axis. This may not only help alleviate depressive symptoms but improve overall health in these patients. Future research may focus on interventions targeting poor sleep quality more specifically, obtaining objective sleep data and recruiting a larger sample to increase power.

## CRediT authorship contribution statement

**Sebastian Laufer:** Writing – review & editing, Writing – original draft, Methodology, Investigation, Formal analysis, Data curation, Conceptualization. **Johannes Bohn:** Methodology, Data curation. **Sinha Engel:** Writing – review & editing, Conceptualization. **Hannah Klusmann:** Writing – review & editing. **Nadine Skoluda:** Writing – review & editing, Formal analysis. **Urs M. Nater:** Writing – review & editing, Formal analysis. **Christine Knaevelsrud:** Writing – review & editing, Supervision, Conceptualization. **Sarah Schumacher:** Writing – review & editing, Supervision, Methodology, Conceptualization.

## Declaration of competing interest

The authors declare that they have no known competing financial interests or personal relationships that could have appeared to influence the work reported in this paper.
