# Supplementary material for: Sleep quality and the biological stress system during an internet-based intervention for major depressive disorder
Source: Compr Psychoneuroendocrinol. 2025 Aug 12;24:100314. doi: 10.1016/j.cpnec.2025.100314 (PMC12395157; doi:10.1016/j.cpnec.2025.100314)
Supplement: Multimedia component 1 [file mmc1.docx]

**Appendix**

**Cortisol Assessment List (CoAL)**

| **Basal cortisol secretion – Reporting of sampling procedures** | | | |
| --- | --- | --- | --- |
| Item | Definition |  | |
| 1 | **Providing instructions stressing the importance of proper sample collection, storage and adherence to the sampling protocol** | - **Considered** | **Notes:** |
| 2 | **Objectively monitoring sampling time points, using time-stamped containers, automatic blood sampling etc.** | ** Considered** | **Notes:** |
| 3 | **Recording exact sampling time points, using logbook, in-person monitoring etc.** | - **Considered** | **Notes:** |
| 4 | **Recording exact wake up time, using logbook, in-person monitoring etc.** | - **Considered** | **Notes:** |
| 5 | **Repeated sampling over at least two consecutive days** | - **Considered** | **Notes:** |
| 6 | **Capturing the cortisol awakening response (i.e. at least one waking sample and a second sample shortly thereafter)** | - **Considered** | **Notes:** |
| 7 | **Collecting an awakening sample** | - **Considered** | **Notes:** |
| 8 | **Fixed sampling time points either related to clock times or to wake up time** | - **Considered** | **Notes:** |
| 9 | **Capturing the diurnal slope using at least 5 sampling points** | - **Considered** | **Notes:**  5 samples including the waking sample |
| **Items specific to assessment of the cortisol awakening response (CAR)** | | | |
| 1 | **Providing instructions to refrain from snoozing or dozing off** | - **Considered** | **Notes:** |
| 2 | **Objectively monitoring wake up time using polysomnography, wristbands etc.** | ** Considered** | **Notes:** |
| 3 | **Obtaining at least three samples to capture the CAR** | ** Considered** | **Notes:** |

**Principal component analysis of the short-term sleep quality indicator (STSQ)**

The full model included the following variables, which were averaged over both assessment days:

1. Sleep quality
2. Sleep efficiency
3. Sleep deficit
4. Times waking up during the night

**Results**

|  | PC1 | PC2 | PC3 | PC4 |
| --- | --- | --- | --- | --- |
| Eigenvalues | 1.664 | 1.274 | 0.542 | 0.520 |
| Standard deviation | 1.290 | 1.129 | 0.736 | 0.721 |
| Proportion of Variance | 0.416 | 0.319 | 0.136 | 0.130 |
| Cumulative Proportion | 0.416 | 0.735 | 0.870 | 1.000 |

Scree plot of explained variance


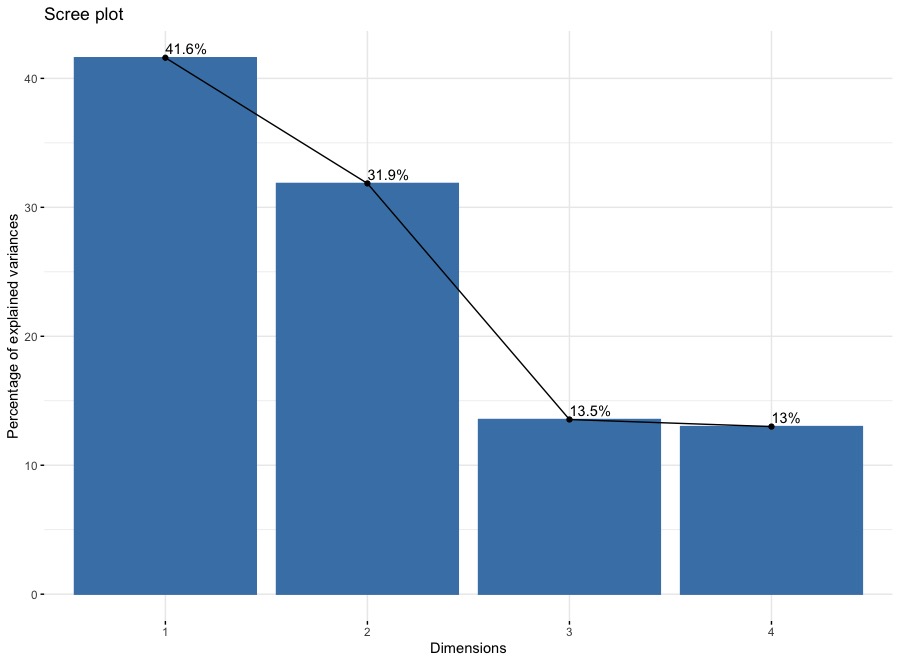


The final model included the following variables:

1. Sleep quality
2. Sleep efficiency
3. Sleep deficit

**Results**

|  | PC1 | PC2 | PC3 |
| --- | --- | --- | --- |
| Eigenvalues | 1.663 | 0.808 | 0.529 |
| Standard deviation | 1.290 | 0.8991 | 0.727 |
| Proportion of Variance | 0.554 | 0.270 | 0.176 |
| Cumulative Proportion | 0.554 | 0.824 | 1.000 |

Scree plot of explained variance


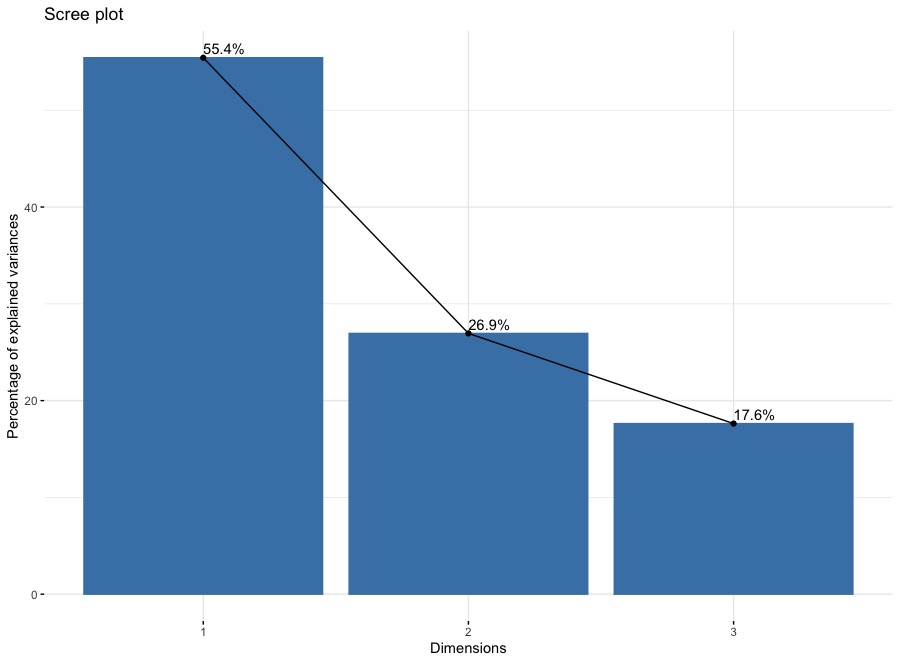


| **Table 2** **extension**  *Analysis parameter values over time*  **sCort:** salivary cortisol; **sAA:** salivary alpha-amylase; **CAR:** Cortisol awakening response; **AUC_g_**: Area under the curve with respect to ground; **AAR:** Alpha-amylase awakening response; **T0:** Pre-intervention assessment time point; **T1:** Mid-intervention assessment time point; **T2:** Post-intervention assessment time point | | | | |
| --- | --- | --- | --- | --- |
| **Variable** |  | **T0** | **T1** | **T2** |
| *CAR*  *M (SD)* |  | 0.16 (0.15) | 0.16 (0.10) | 0.14 (0.13) |
| *sCort AUC_g_*  *M (SD)* |  | 1151 (499) | 1252 (558) | 1129 (484) |
| *sCort slope*  *M (SD)* |  | -0.21 (0.17) | -0.22 (0.13) | -0.21 (0.12) |
| *AAR*  *M (SD)* |  | -3.79 (3.31) | -3.59 (3.17) | -3.71 (2.68) |
| *sAA AUC_g_*  *M (SD)* |  | 84277 (57467) | 86211 (56391) | 90456 (57406) |
| *sAA slope*  *M (SD)* |  | 0.337 (7.54) | 1.97 (7.18) | 2.16 (9.51) |
